# Functional and Muscular Adaptations in an Experimental Model for Isometric Strength Training in Mice

**DOI:** 10.1371/journal.pone.0079069

**Published:** 2013-11-13

**Authors:** Karsten Krüger, Denise K. Gessner, Michael Seimetz, Jasmin Banisch, Robert Ringseis, Klaus Eder, Norbert Weissmann, Frank C. Mooren

**Affiliations:** 1 Department of Sports Medicine, Institute of Sports Sciences, Justus-Liebig-University Giessen, Giessen, Germany; 2 Institute of Animal Nutrition and Nutrition Physiology, Justus-Liebig-University Giessen, Giessen, Germany; 3 Universities of Giessen and Marburg Lung Center (UGMLC), Excellence Cluster Cardiopulmonary System (ECCPS), Member of the German Center for Lung Research (DZL), Giessen, Germany; Universidad Pablo de Olavide, Centro Andaluz de Biología del Desarrollo-CSIC, Spain

## Abstract

Exercise training induces muscular adaptations that are highly specific to the type of exercise. For a systematic study of the differentiated exercise adaptations on a molecular level mouse models have been used successfully. The aim of the current study was to develop a suitable mouse model of isometric strength exercise training characterized by specific adaptations known from strength training. C57BL/6 mice performed an isometric strength training (ST) for 10 weeks 5 days/week. Additionally, either a sedentary control group (CT) or a regular endurance training group (ET) groups were used as controls. Performance capacity was determined by maximum holding time (MHT) and treadmill spirometry, respectively. Furthermore, muscle fiber types and diameter, muscular concentration of phosphofructokinase 1 (PFK), succinate dehydrogenase (SDHa), and glucose transporter type 4 (GLUT4) were determined. In a further approach, the effect of ST on glucose intolerance was tested in diabetic mice. In mice of the ST group we observed an increase of MHT in isometric strength tests, a type II fiber hypertrophy, and an increased GLUT4 protein content in the membrane fraction. In contrast, in mice of the ET group an increase of VO_2max_, a shift to oxidative muscle fiber type and an increase of oxidative enzyme content was measured. Furthermore strength training was effective in reducing glucose intolerance in mice fed a high fat diet. An effective murine strength training model was developed and evaluated, which revealed marked differences in adaptations known from endurance training. This approach seems also suitable to test for therapeutical effects of strength training.

## Introduction

Human body is a highly dynamic and plastic system with respect to adaptation responses to exercise. Depending on the type of exercise strain tissues demonstrate an improvement of either their functional, structural and/or metabolic properties. Thereby pure endurance and strength training stimuli represent two important extremes which are implemented into many training regimes both in high performance and recreational sports [1], [2].The endurance phenotype is characterized by a high resistance against fatigue and a quick recovery after exercise. Furthermore, muscles are enriched with slow twitch fibers (type I fibers), and are abundantly furnished with oxidative enzymes and mitochondria allowing prolonged bouts of endurance activities [3], [4]. In contrast, resistance training athletes are hypermuscular yet lean. They exhibit an increased maximal strength and are able to perform short term high intensity exercise programs. Their muscles are enriched with fast glycolytic fibers that express myosin heavy chain (MHC) proteins type IIa and IIx. These fibers express primarily glycolytic enzymes to enhance glucose utilization and ATP generation. However, these muscles have only a limited resistance against fatigue [3], [5].

Beyond athletes performance exercise training has become an important life style factor known to be effective in prevention and therapy of many wealth-related diseases, such as many metabolic and cardiovascular diseases [6], [7]. While recreational training recommendations focused for a long time on endurance exercise only recently other motoric properties such as strength have been evaluated in more detail. As many effects of exercise training in prevention and therapy are based on functional and structural muscle adaptation, recent works demonstrated an important role for both endurance as well as for strength/resistance training in prevention and therapy of obesity and type I diabetes [8], [9]. Accordingly, there are two different approaches to address metabolic dysfunction in skeletal muscle: endurance exercise training promotes an increased glucose and fatty acid oxidation and mitochondrial biogenesis and resistance exercise training to increase muscle mass, basic metabolic rate and activity of glycolytic fibers [9], [10].

For a systematic study of the exercise effects on a molecular level, animal models have been used successfully. While endurance exercise models have been established and widely reported in the literature, the use of resistance/strength exercise models is still rare [11]. Klitgaard (1988) and Nicastro *et al.* (2012) described rodent models of resistance exercise which resulted in specific adaptations known from humans. However, these experimental approaches showed either some limitations in extrapolations to human conditions or used complex training apparatus [12], [13]. Likewise, recently published models used loaded wheel running which can be characterized more as a type of combined resistance-endurance training [14].Therefore, the aim of the current study was to develop a murine resistance/strength exercise model which can be characterized by simple handling and adequate adaptation responses. We hypothesized that this regular isometric strength training resulted in specific functional, structural, and biochemical adaptations known from resistance exercise training in humans and which would be clearly distinguishable to endurance training. Knowing that resistance exercise in humans is also effective in therapy of diabetes we further hypothesized that this training method is reducing glucose intolerance in a mouse model of obesity.

## Methods

### Ethics Statement

This study was carried out in strict accordance with the recommendations in the Guide for the Care and Use of Laboratory Animals. All animals were housed, cared for, and the protocol was approved by the Animal Welfare Officer of the Justus-Liebig-University and the Regierungspräsidium Giessen (No. 94/2010).

### Animals and experimental groups

30 male C57BL/6N mice (aged 10–12 weeks) were assigned to a strength training group (ST),an endurance training group (ET), and a control group (CT).The initial body weights can be found in table 1. Mice were housed 4–6 per cage at 21±1°C in standard cages with free access to food and tap-water and were bred at the animal facility of the Department of Sports Medicine (Justus-Liebig-University, Giessen).

**Table 1:** Body weight of mice of CT, ET and ST groups before (pre), during (5 weeks) and at the end (10 weeks) of the interventions.

Body weight (g)	pre	5 weeks	10 weeks
CT	23.1±0.4	26.4±0.4#	27.1±0.5#
ET	22.8±0.4	25.2±1.0#	25.8±0.5#
ST	22.2±0.5	25.5±0.6#	26.1±0.2#

Data are given as means ± SEM, for all figures # indicates significant differences to pre training (p<0.05).

In order to investigate effects of exercise training on diabetes and obesity, 3 additional groups were established (3 groups, n = 10 each, same age and weight as other groups): High fat control group (hfCT), a high fat endurance training (hfET) and a high fat strength training (hfST) group. High fat diet groups were switched for 10 weeks on a lard diet to induce obesity as previously described [15].

### Exercise training protocols

**Figure 1:**
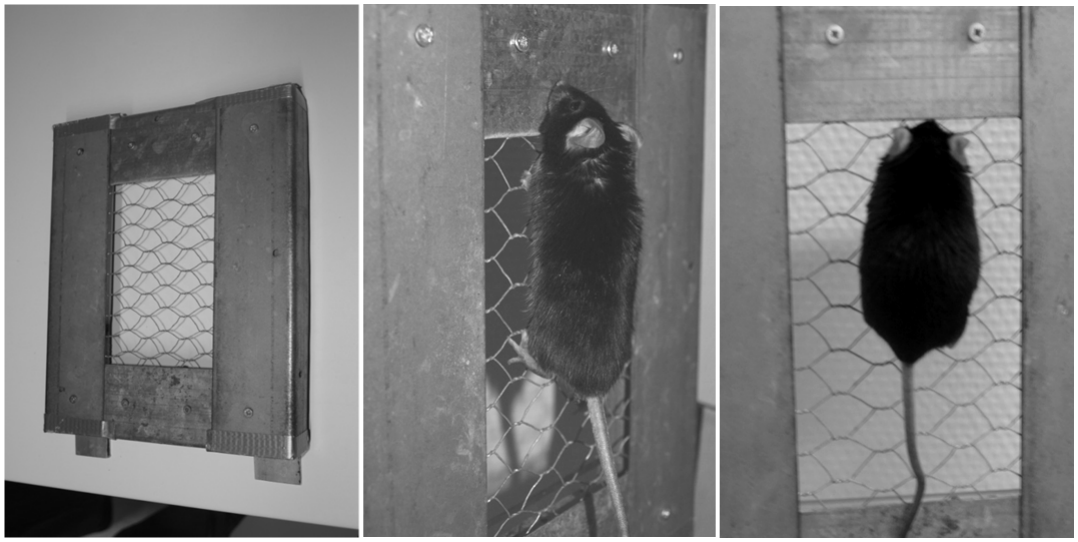
External view of the equipment for strength training and mice during strength exercise training.

All animals were housed on a reverse light-dark cycle (lighting from 21:00 to 09:00 h). Isometric strength training was performed by the following experimental set up. A 8×5 cm hole was cut in an aluminium (AE) plate and covered with a metal wire mesh. The wire had a maximum diameter of 1 mm to provide mice a strong grip. The mice gripped with their front and their back paws on horizontal wires of the metal mesh and the plate was placed in a vertical position (Fig. 1). Meanwhile the orientation of the mice was holding head up. The plain surface of the AE plate prevented a further movement away. Strength training was performed for 5 times/week for 3 minutes and 3 series. Break between each series was 1 minute.

Mice of the ET group performed a treadmill run for 35 min/day at 12 m/min, 5 times per week, for 10 weeks. Running speed was 0.27±0.05 m/s corresponding to about 80% of VO_2max_.

### Functional strength and endurance testing

In order to determine isometric strength we used the same experimental set up like in the training protocol (maximum holding time  =  MHT). Briefly, the mice gripped with their paws on wires of the metal mesh and the plate was placed in a vertical position. Time was measured until mice released both back paws from the wire.

Aerobic capacity was determined by using a treadmill spirometry as previously described [16], [17]. Briefly, after a short acclimatization maximal oxygen consumption (VO_2max_) and maximal running speed (V_max_) of mice were tested at least 4 days before starting the experiments. After 10 min of acclimatization in the treadmill chamber, mice performed a continuous, progressive exercise test until exhaustion. The test uptake started at 0.15 m/sec, every 3 min the speed was increased by 0.05 m/sec VO2_max_ and V_max_ were analyzed.

### Fiber typing and determination of muscle diameter

Serial cross sections (20 µm thick) of m. rectus femoris, m. soleus and m. gastrocnemius were cut on a cryostat microtome at −25°C. Muscle cross sections were mounted on cover slips and stained for myosin ATPase (mATPase) with acid pre-incubation using a modified method according to Hämäläinen and Pette [18].Briefly, sections were pre-incubated for 5 min in sodium acetate (54.3 mM) – sodium barbital (32.6 mM) solution adjusted with HCl to pH 4.6. After washing, the sections were incubated for 30 min at 37°C in substrate solution (2.7 mM ATP, 100 mMglycin, 54 mM CaCl_2_, 100 mMNaCl, pH adjusted to 9.6). After incubations in 11 mM CaCl_2_ and 2% CoCl_2_, a black insoluble compound was developed in 1% ammonium sulfide for 50 s. Therefore, “black” type I fibers can be distinguished from “grey” type II fibers. After washing with distilled water, the sections were analyzed by light microscopy (Leica DMI 6000B,Leica Microsystems, Wetzlar Germany) for calculating the type I and type II fiber percentages and measurement of average muscle fiber diameters by using Leica Application Suite software and Leica QWin (Leica Microsystems,Wetzlar Germany).

### RNA isolation, cDNA synthesis and real-time polymerase chain reaction

Preparation of tissue and gene expression analysis was performed as previously described [19]. Total messenger RNA was isolated from homogenized mouse muscles (m. rectus femoris, m. gastrocnemius, m. soleus) using RNeasy Mini Kits (Qiagen, Hilden, Germany) according to the manufacturer's instructions. 1 µg of isolated RNA each was converted to cDNA by reverse transcription using the iScriptcDNA Synthesis Kit (BioRad, Munich, Germany). The conditions for the reverse transcription were as follows: 1 cycle at 25°C for 5 min; 1 cycle at 42°C for 30 min; 1 cycle at 85°C for 5 min.

Relative quantification of glucose transporter type 4 (GLUT4), phosphofructokinase (PFK) and succinate dehydrogenase subunit A (SDHa) was performed by real-time PCR with the iQ SYBR Green Supermix according to the manufacturer's instructions (BioRad, Munich, Germany). Per reaction, a 25 µL mixture was used containing 12.5 µL iQ SYBR Green Supermix, 0.5 µL forward and reverse primer, 9.5 µL sterile water, and 2 µL of the 1∶5 diluted complementary DNA template. A negative control (nontemplate control) was performed in each run. The Real-time PCR experiments were performed with a Mx3000P (Stratagene, Heidelberg, Germany) under the following conditions: 1 cycle at 95°C for 10 min, then 40 cycles at 95°C for 10 s, 59°C for 10 s, 72°C for 10 s, followed by a dissociation curve. The intron-spanning primers were designed by using sequence information from the NCBI database. The Ct values were normalized to the endogenous control (Porphobilinogendeaminase, PBGD).

Following primers were used:

PhosFK_mouse_F 5′-AGGGGAAGGGCATCTTTGAT-3′

PhosFK_mouse_R 5′-AGTCAGGGGTGTTGGCAAAG-3′

GLUT4_mouse_F 5′-CGGGTTTCCAGCAGATCG-3′

GLUT4_mouse_R 5′-GGGCATTGATAACCCCAATG-3′

SDHa_mouse_F 5′-GCCTGGTCTGTATGCCTGTG-3′

SDHa_mouse_R 5′-CCGATTCTTCTCCAGCATTTG-3′

PBGD_mouse_F 5′-GGGAACCAGCTCTCTGAGGA-3′

PBGD_mouse_R 5′-GAATTCCTGCAGCTCATCCA-3′

### Glucose tolerance test

Glucose tolerance was estimated as described previously (31). Briefly, following a 12-h fasting period, tail vein blood was taken before (T0), 30 min after (T1), 60 min after (T2), and 2 h after (T3) intraperitoneal application of 2 g glucose (dissolved in phosphate-buffered saline) per kilogram body weight. Blood glucose concentration was measured using a standard glucometer (Roche Diagnostics, Mannheim, Germany).

### Immunoblot analysis

Frozen muscles were homogenized with a homogenizer (Precellys 24, Peqlab, Erlangen, Germany). The respective tissues were put in 2 ml tubes containing ceramic beads and homogenization buffer containing protease inhibitors (from ab65400, Abcam) and homogenized (6800 rpm, 2×30 s, each) followed by additional homogenization using syringes with 20 G needles. Such homogenized tissues were incubated 30 min on ice. The separation of cytosolic and total membrane proteins was performed with the Membrane Protein Extraction Kit (ab65400, Abcam) according to the manufacturer′s instructions. The homogenate was centrifuged at 700×g for 10 min at 4°C to remove unbroken cells and nuclei. The supernatant was centrifuged at 10,000×g at 4°C to pellet the total cellular membrane fraction which, according to the protocol, contained proteins from both plasma membrane and cellular organelle membrane. The resulting pellets were suspended in 75 µl PBS containing 1 mM sodium vanadate, 0.1 mM phenylmethylsulphonyl fluoride (PMSF), 40 µl/ml protease-inhibitor mix complete® (25× stock solution, Roche, Mannheim, Germany) and used as total membrane fractions (TMF). The supernatant represented the cytosol fraction (CF). Protein concentrations in the TMF and CF were determined by the bicinchoninic acid protein assay kit (Interchim, Montluçon, France) with BSA as standard and frozen at −80°C. Per sample, 20 µg from both fractions were separated on 12% SDS-PAGE and electrotransferred to a polyvinylidene fluoride membrane (Pall Corporation, Dreieich, Germany) by the semidry-blotting method. Equal loading of proteins was demonstrated byPonceau S (Carl Roth, Karlsruhe, Germany) staining, routinely used as loading control[20], [21].The membrane was washed for 5 min with wash buffer (20 mMTris-Cl, pH 7.5, 150 mMNaCl, 0.1% (v/v) Tween 20) and subsequently blocked in 6% (w/v) nonfat dry milk powder dissolved in wash buffer at room temperature. Incubation with a diluted primary antibody against GLUT4 (polyclonal rabbit anti-GLUT4 antibody; Abcam, Cambridge, UK) and Transferrin (membrane-specific protein) (monoclonal mouse anti-Transferrin antibody, Invitrogen, Karlsruhe, Germany) was performed overnight at 4°C. After washing several times with wash buffer, a horseradish peroxidase-conjugated secondary antibody (anti-rabbit_W401B and anti-mouse_W402B, respectively; Promega, Mannheim, Germany) was applied for 1 h at room temperature. After washing the membrane, visualization was carried out using the enhanced chemiluminescence kit (ECL, Amersham, Braunschweig, Germany) and X-ray photo film (Kodak, Stuttgart, Germany). The signal intensities of specific bands were detected with a Bio-Imaging system (ChemiDoc XRS+, Biorad, Munich, Germany) and quantified using ImageLab® software (Biorad, Munich, Germany).Ponceau S staining of the membranes was converted to a gray scale.

### Statistical analysis

Data are means ± SEM, unless indicated otherwise in the figure legends. Differences between the points of time and groups were analysed by repeated measures ANOVA followed by Bonferroni's Multiple Comparison Test. In all cases, p<0.05 was accepted as being significant.

## Results

### Body weight and exercise performance

Body weight changes were similar for both exercise and control group. Body weight increased significantly between pre and 5 weeks of exercise training, while no further increase was observed between 5 weeks and 10 weeks of training (Table 1).

**Figure 2:**
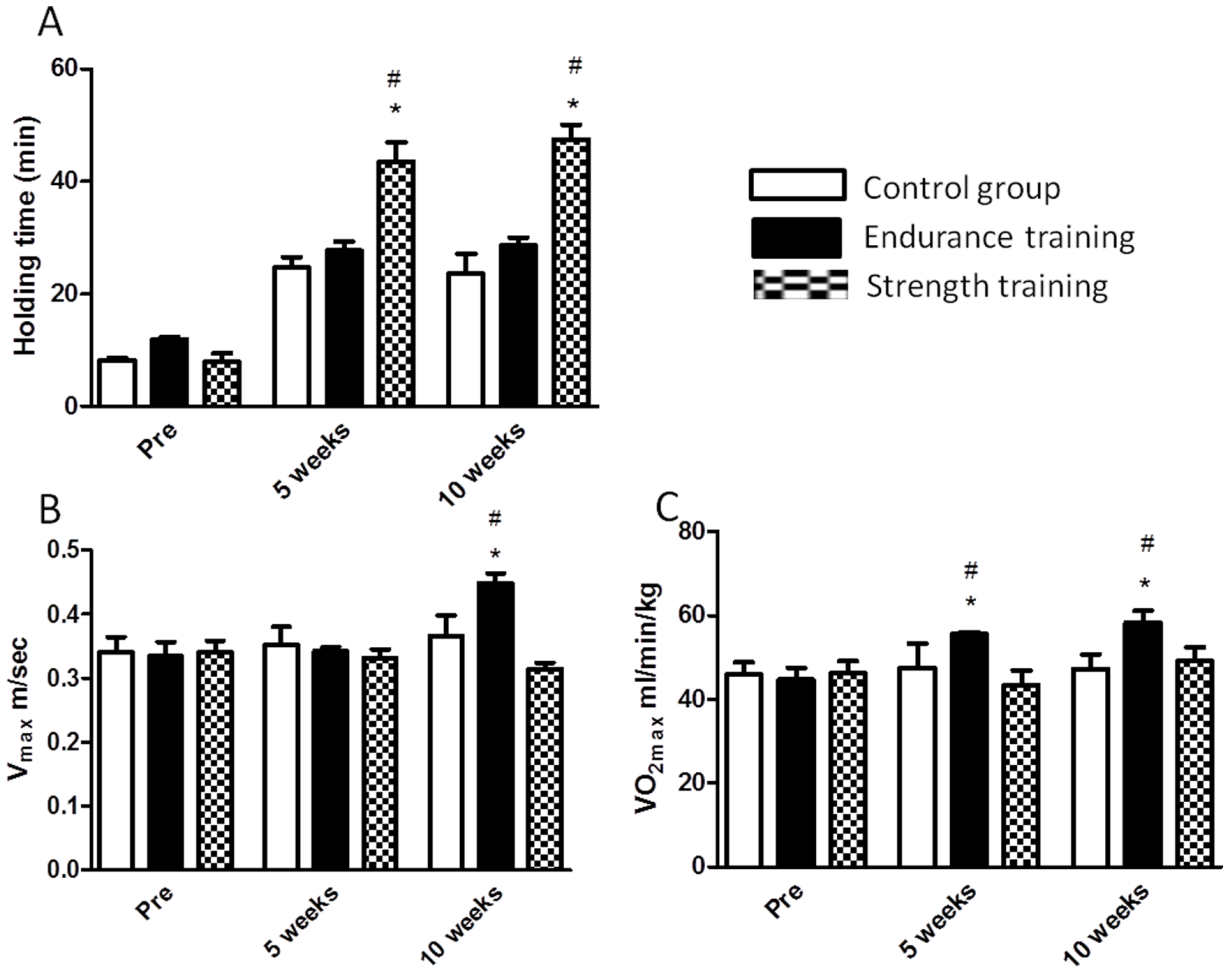
Holding time (A), V_max_ (B) and VO_2max_(C) of CT, ET and ST groups before (pre), during (5 weeks) and at the end (10 weeks) of the interventions. Data are given as means ± SEM, for all figures * indicates significant differences to pre training value of same group, # indicates differences from both other groups same point of time (p<0.05).

MHT increased significantly only in mice from the ST group between pre and 5 weeks as well as 5 weeks and 10 weeks (p<0.05, Fig. 2A). Here, no significant changes were observed neither in the CT nor the ET group. In contrast, no changes of V_max_ were observed in the ST group, while we found an increase in the ET group between pre and 10 weeks of training (Fig. 2B). Similarly, VO_2max_did not change neither in the ST nor in the CT group, while it increased significantly in the ET group between pre and 5 weeks as well as between 5 weeks and 10 weeks (p<0.05, Fig. 2C).

### Muscle fiber types

**Figure 3:**
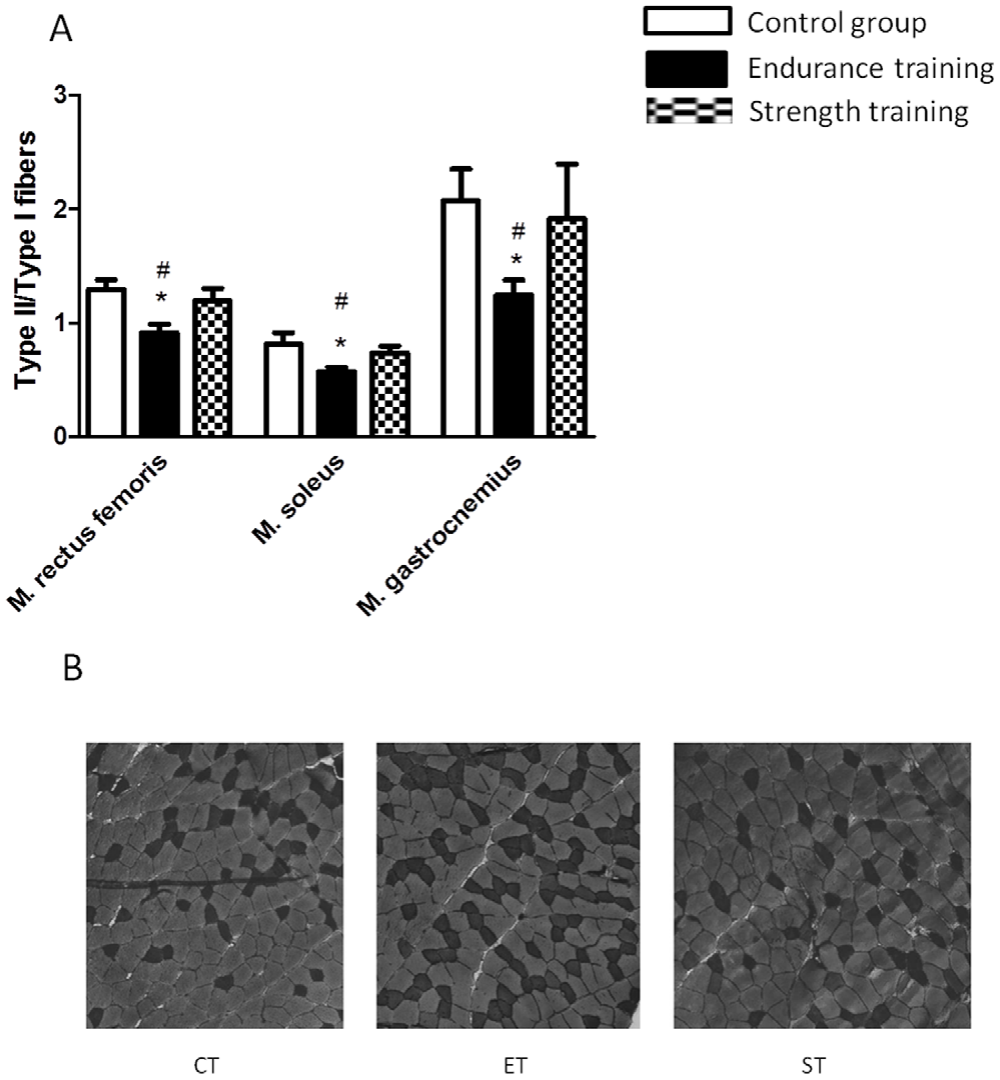
Relation of type II to type I fibers in m. rectus femoris, m. soleus, and m. gastrocnemius in CT, ET and ST groups after training (A). Figure 2B shows representative pictures from fiber typing in different groups. Data are given as means ± SEM, for all figures * indicate significant differences to CT group (p<0.05), # indicates differences to ST group (p<0.05).

There was a different fiber type distribution between m. rectus femoris, m. soleus, and m. gastrocnemius in the control group as indicated by the ratio of type II/type I fibers. The ratio was found to be highest in m. gastrocnemius, while the proportion of type I fibers continuously increased from m. rectus femoris to m. soleus (p<0.05, Fig. 3).

We found no fiber type changes in muscles from the ST group. In contrast, in all three muscles, endurance training resulted in a decrease of the ratio type II/type I fibers compared to CT indicating a proportional increase of type I fibers (p<0.05, Fig. 3).

### Muscle fiber thickness

**Figure 4:**
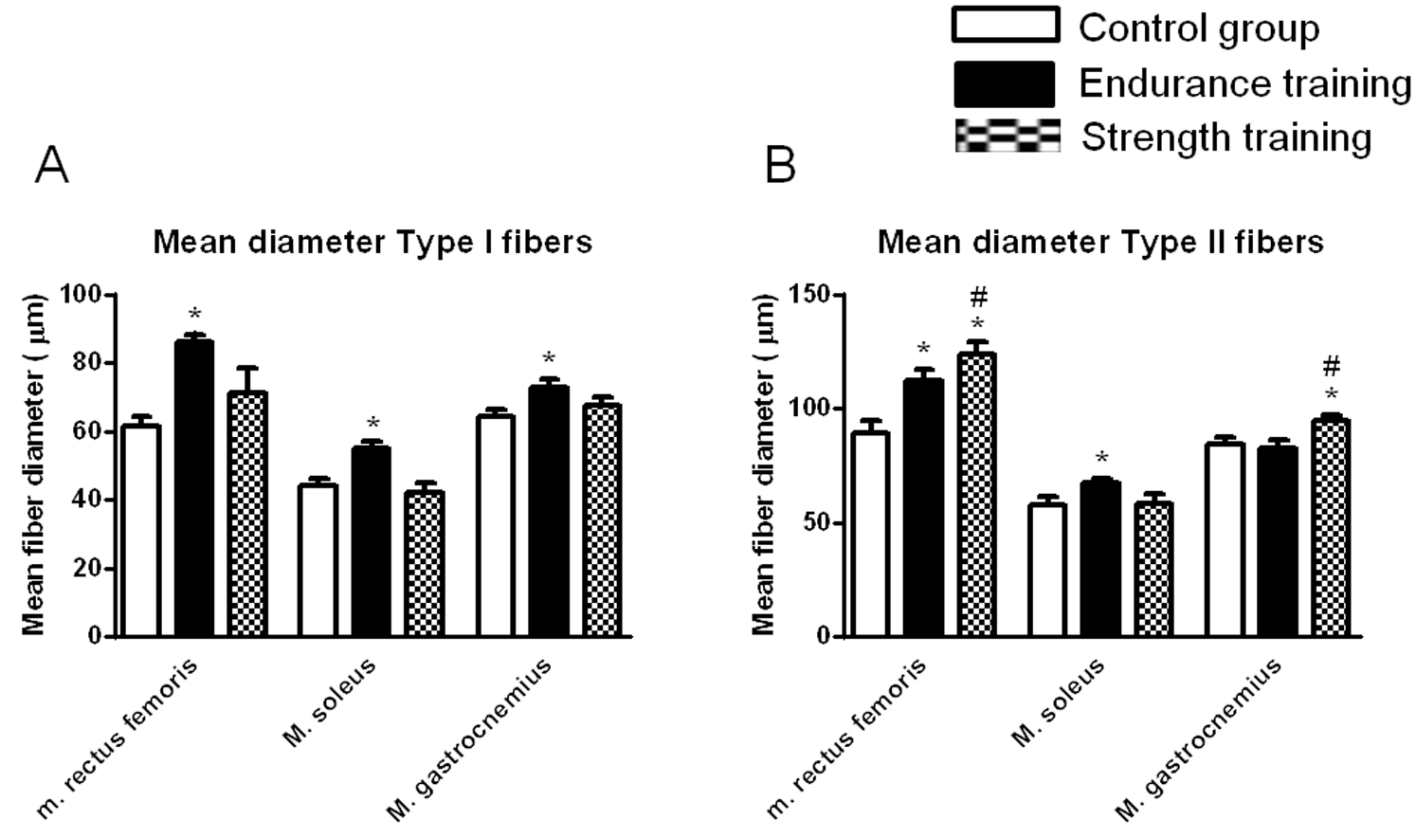
Mean diameter of type I fibers (A) and type II fibers (B) of m. rectus femoris, m. soleus, and m. gastrocnemius in CT, ET and ST groups after training. Data are given as means ± SEM, for all figures * indicates significant differences between groups (p<0.05).

In addition to muscular fiber type composition the fiber cross section was measured. In m. rectus femoris, m. soleus, and m. gastrocnemius, exercise training interventions were followed by differential changes in fiber thickness depending on fiber type. Strength training increased the diameter of type II fibers in m. rectus femoris as well as m. gastrocnemius significantly against both other groups, while no changes occurred in m. soleus. Interestingly, a slight increase of type II fiber thickness in m. rectus femoris and m. soleus was also found after endurance training. Regarding type I muscle fibers, no changes in mean diameter in any muscle was found in the ST group, while an increase was found in the ET group (p<0.05, Fig. 4).

### Muscle fiber enzymes

**Figure 5:**
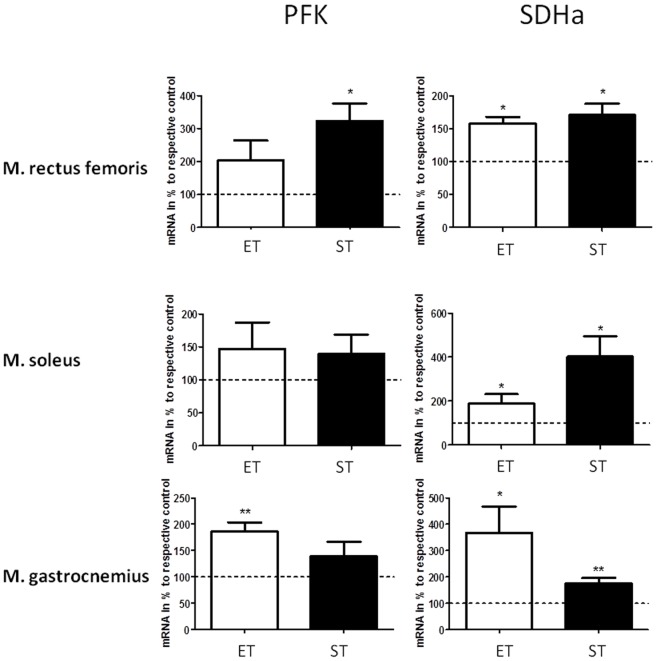
Relative expression of mRNA of PFK and SDHa in m. rectus femoris, m. soleus, and m. gastrocnemius of ET and ST group relative to respective control. Values of CT group were set to 100%. */** indicates significant differences to control (p<0.05, p<0.01).

Changes in muscle phenotype were accompanied by changes of muscle enzyme expression. We found a significant increased mRNA content of PFK in m. rectus femoris of mice from the ST group, while no changes were observed in ET group. No changes of PFK expression were found in soleus muscle. In the m. gastrocnemius expression of PFK was enhanced in the ET group only while strength training had no effect (p<0.05, Fig. 5).

Focusing on oxidative enzymes, a significant up-regulation of SDHa mRNA was found in all three muscles investigated as well as after both training regimes (p<0.05, Fig. 5).

### Expression of GLUT4 mRNA/protein

**Figure 6:**
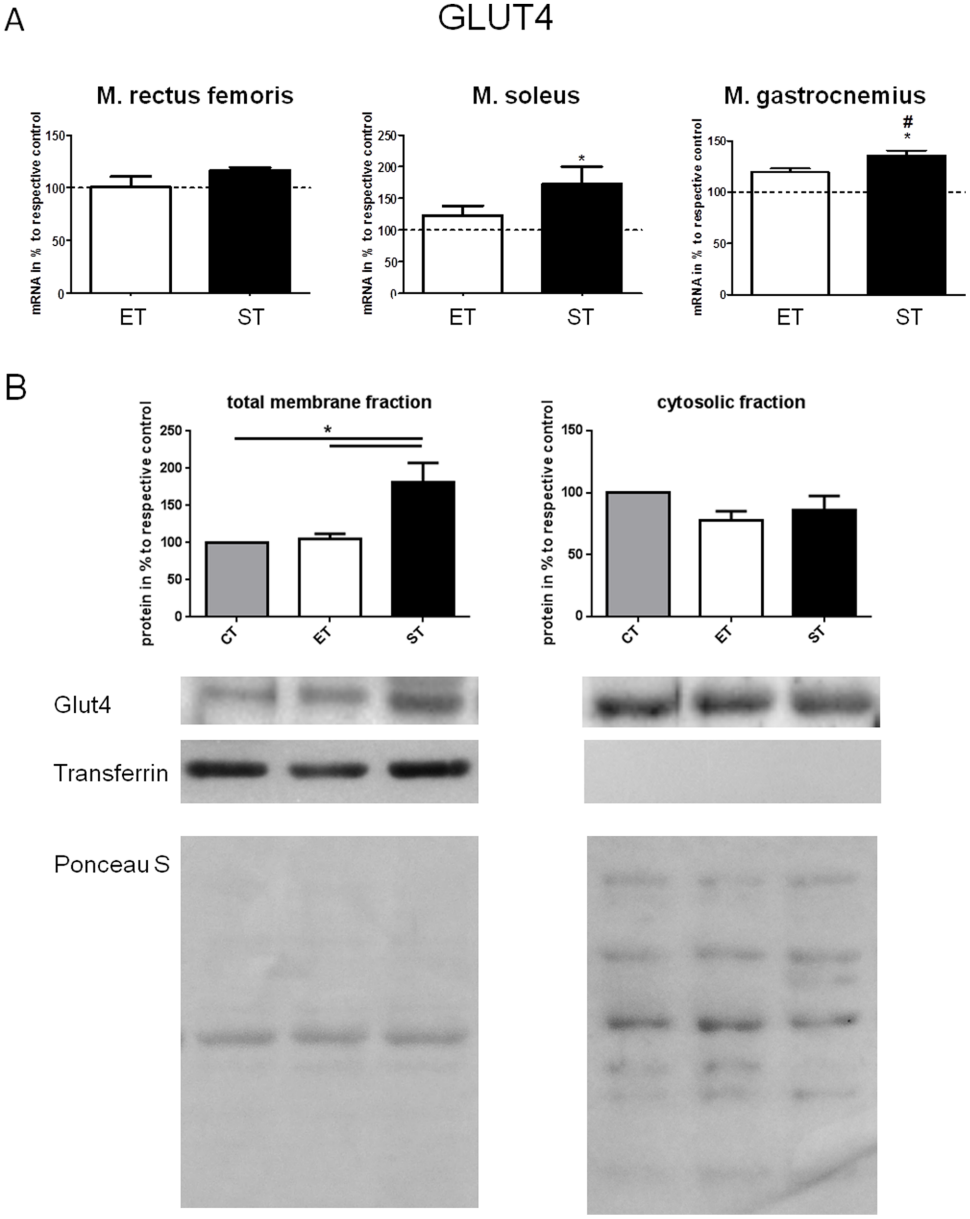
A. Relative expression of mRNA of GLUT4 in m. rectus femoris, m. soleus, and m. gastrocnemius of ET and ST group relative to respective control. Values of CT group were set to 100%. */^#^ indicates significant differences to control and ET respectively (p<0.05). B. Expression of GLUT4 proteins in total membrane and cytosolic fraction of m. rectus femoris in mice of the CT, ET and ST group. Values of CT group were set to 100%. Gels were spliced to present the results from the strength training (ST) in the consecutive sequence according to figures. * shows significant differences as indicated (p<0.05). Ponceau S staining demonstrates equal loading of proteins. Data are presented as mean ± SEM.

Next the expression of the substrate transporter GLUT4 was investigated. Strength training enhanced GLUT mRNA in m. soleus and m.gastrocnemius significantly (p<0.05, Fig. 6a), while after endurance training no change in transcription was found. Similarly, GLUT4 protein content was only significantly enhanced in the membrane fraction of rectus muscle in the ST group after training (p<0.05, Fig. 6B).

### Glucose handling

**Figure 7:**
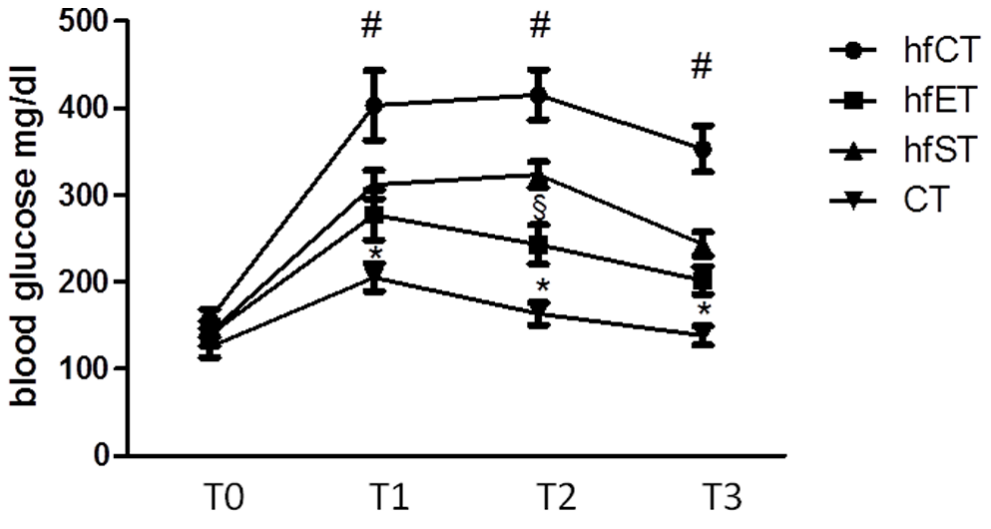
Blood glucose levels after at T0before (T0), 30 min after (T1), 60 min after (T2), and 2 h after (T3) intraperitoneal application of 2 g glucose. # indicates significant differences of hfCT to all other groups, * indicates significant differences of CT to all other groups, § indicates significant differences to ET group (p<0.05).

**Figure 8:**
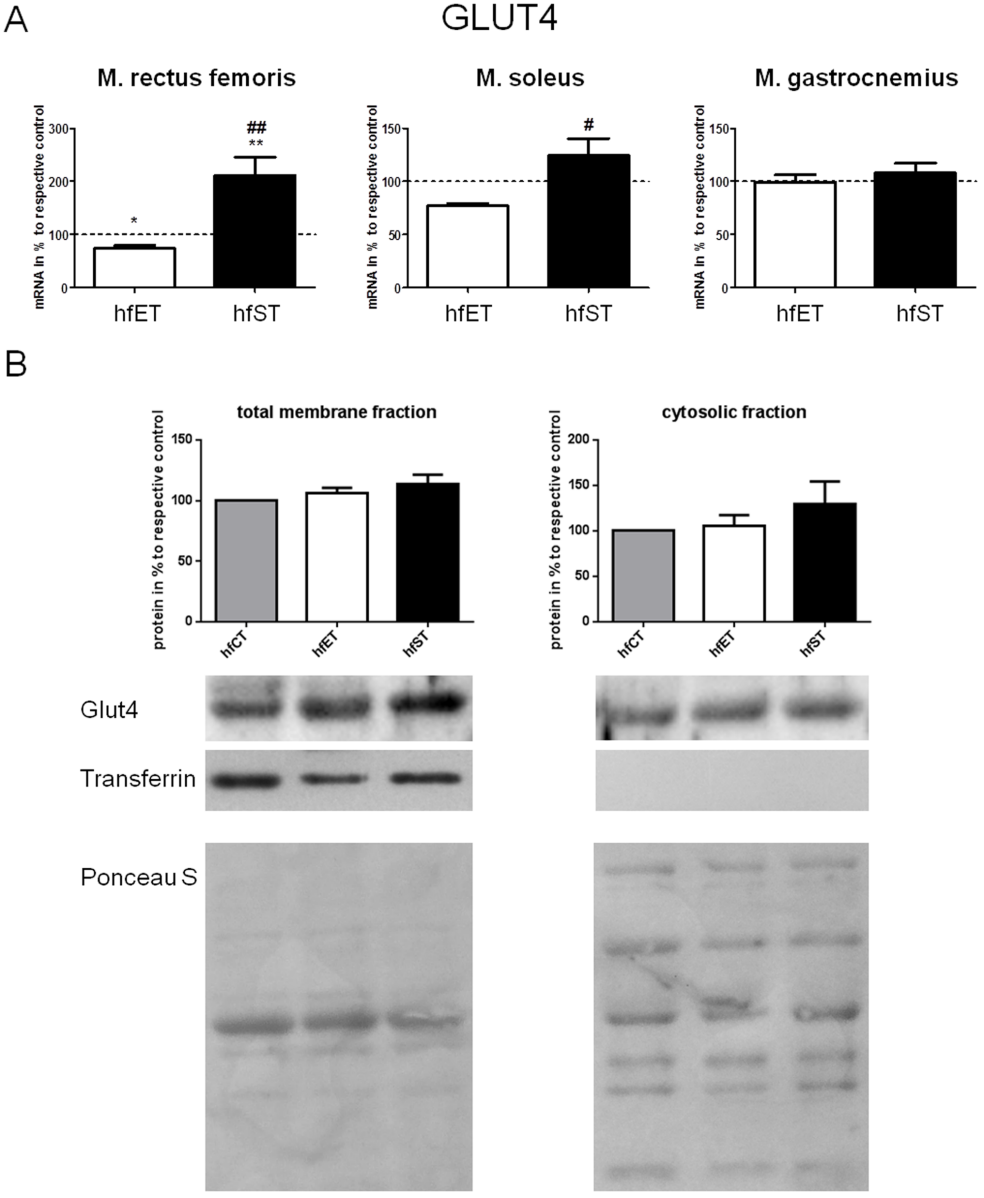
A. Relative expression of mRNA of GLUT4 in m. rectus femoris, m. soleus, and m. gastrocnemius of hfET and hfST group relative to respective control. Values of CT group were set to 100%. */** indicates significant differences to hfST (p<0.05/p<0.01). B. Expression of GLUT4 proteins in total membrane and cytosolic fraction of m. rectus femoris in mice of the hfCT, hfET and hfST group. Gels were spliced to present the results from the hf strength training (hfST) in the consecutive sequence according to figures.*shows significant differences as indicated (p<0.05).Ponceau S staining demonstrates equal loading of proteins. Data are presented as means ± SEM.

In order to evaluate the effect of the two training modes on glucose handling, a murine diabetes model was used. Mice which were chronically fed a high-fat diet showed a dysregulation of blood glucose levels after an intraperitoneal glucose tolerance test. While blood glucose levels in control mice were about 200 mg/dl in the maximum, values of approximately 400 mg/dl were reached for the high-fat diet animals (p<0.05, Fig. 7). Exercising mice parallel to application of the high-fat diet resulted in significant lower glucose levels than for the sedentary animals indicating an improved glucose tolerance. Both training regimes were similar effective in improving glucose handling except time point T2 (p<0.05, Fig. 7). Finally, the GLUT4 expression was investigated in the diabetes/training model. Again, GLUT4 mRNA was enhanced in m. rectus femoris and m. soleus only in the ST group, while endurance training induced a slight decrease of GLUT mRNA at least in m. rectus femoris (p<0.05, Fig. 8A). In contrast, both training regimes did not show significantly enhanced GLUT4 expression on a protein level in the high fat diet, neither in the respective cytosolic, nor in the membrane fractions. While ST resulted in an enhanced GLUT4 expression by trend in the membrane and cytosolic fraction, ET did not affect GLUT4 expression in both fractions(p<0.05, Fig. 8B).

## Discussion

In the current study the effects of a novel isometric strength training model on functional parameters and skeletal muscle adaptations in mice were examined. The main findings were that mice demonstrated specific adaptations including a prolonged holding time in MHT testing, a type II fiber hypertrophy, and an increased GLUT4 translocation in muscle membranes. The specificity of these adaptation responses to strength exercise was supported by comparison with the alterations induced after endurance training. The therapeutic effects known from strength training in diabetic humans could also be mimicked by demonstrating that strength training was effective in improving glucose handling in a murine high fat diet model.

At first, the advantages of the strength training method should be discussed. The strength training device is characterized by both simple construction and easy manageable which accounts for typical rodent behavior such as gripping and climbing during their habitual movements in standard cages. Therefore, training did not seem to be stressful and did not need fasting, shocking or conditioning compared to other models of strength training. The model applies predominantly static and isometric forces which contrasts to most of the other approaches reported in the literature so far. In addition, it was possible to effectively control the variables holding time, repetitions and rest interval. For future studies, the model can be extended by fixing additional weights to their tails in order to increase strength training intensity.

Regarding exercise performance parameters, it could be clearly demonstrated that the specific training protocols resulted in different changes of motor performance. Whilemice of the ST group increased their holding time approximately 6-fold, no changes were observed in CT or in ET groups. In contrast, mice of the ST group demonstrated no increase of endurance capacity as indicated by measurement of VO_2max_ as well as V_max_.

These results clearly show that the isometric strength training induced specific motoric adaptations without any functional interference with endurance performance. Such an absence of functional overlapping with endurance capacity was one of the requirements in the development of the strength training model since isometric strength training in humans is known to affect parameters of aerobic capacity only marginally [22], [23], [24], [25].

However, in humans, maximum strength is often measured by determination of the one repetition maximum or dynamic strength testing which are both depending on the highest weight which can be moved [26], [27]. Obviously, these types of testing were not applicable with mice. Instead, we used the holding time for quantification of strength capacity. Using this parameter was obviously a limitation in the current study since maximum strength is defined as the greatest amount of force that can be produced in a single exertion [26]. Other studies used weight suspension in vertical climbing rats or weight cylinders [12] which depend on complex instruments making their application to mice difficult. However, intention of the current strength training model was mainly to develop a model which was easy to handle and on the same time able to induce adequate adaptations. Changes in exercise performance capacity are usually a result of changes in muscle fiber types and/or size as the different fiber types have different functional capabilities. In this framework, ATPase activity method is often used as a fundamental method in order to distinguish between oxidative type I and glycolytic type II muscle fibers. Three phenotypically different muscles, which were supposed to be involved in both treadmill locomotion as well as isometric holding, were analyzed: m. rectus femoris, representing a mixed muscle of both oxidative and non-oxidative fibers, m. soleus which has a more oxidative phenotype, and m. gastrocnemius which has a more glycolytic phenotype.

After isometric strength training no muscle fiber type shift was observed which corresponded to most human studies [28]. In contrast, endurance training typically results in an overall shift away from type IIb expressing fibers to a more oxidative phenotype expressing type IIa or type I muscle fibers in human and rodents, which is in line with our results [22], [29], [30].However, differences between type IIa and type IIb were not determined. Composition of muscles with fibers of a more glycolytic phenotype is usually associated with a more anaerobic capacity, while oxidative capacity increases with a higher number of oxidative fibers [30].Although strength training failed to affect fiber type composition, it was able to affect fiber size. In particular, ST training was followed by a significant increase of diameter of type II fibers in m. rectus femoris and m. gastrocnemius, while it didn't affect type I fibers. These results are supported by data from human subjects which showed a skeletal type II fibers muscle hypertrophy after strength training [2]. However, the effect was not observed in m. soleus suggesting that this muscle is not active during strength training. Interestingly, also endurance training seemed to affect muscle fiber size of both fiber types in the murine model. It can be assumed that sedentary mice in common standard cages are generally suppressed in their activity. Therefore, even endurance exercise induces a type II hypertrophy. However, hypertrophy of type II fibers was most pronounced after strength training.

Metabolic shifts of muscles to a more glycolytic or more oxidative phenotype often coincides with differential expression of enzymes. Therefore, expressions of PFK and SDH are often used as metabolic markers for muscle glycolytic or oxidative capacity, respectively [30]. PFK mRNA expression was found to be increased in the m. rectus femoris of the ST group compared to mice from the CT group. PFK is thought to be the speed limiting enzyme in glycolysis which catalyzes the conversion of fructose 6-phosphate and ATP to fructose 1.6-bisphosphate and ADP. Therefore, an increased expression in m. rectus femoris might enhance availability of ATP during anaerobic glycolysis which agreed with our results from strength testing. An increase of PFK activity indicated a specific adaptation to resistance or strength training, which is typically absent after endurance exercise [31], [32]. Surprisingly, an increase of PFK mRNA expression in m. gastrocnemius of the ET group was observed. Although this result seemed to contradict our expectations, other studies found also a nearly doubled activity of PFK in this muscle after endurance training [8]. Therefore, it can be assumed that an increased glycolytic metabolism is also needed for an increased aerobic metabolism. A significant increase of SDH mRNA expression was found after both strength as well as endurance training in all muscles. Increased SDH activity is known to occur after endurance training, while some studies also found also increased SDH levels in muscles after resistance exercise [33], [34]. These findings agreed with our data and indicated, that strength training also partially affects muscles oxidative capacity which might be a prerequisite for strength endurance [14], [30]. Despite the functional differences between both training models this point seems to address some overlapping of metabolic adaptations.

In addition to muscle fiber enzyme furnishment the expression of the substrate transporters GLUT4 was measured on both mRNA and protein level. Here it was demonstrated that strength training enhanced transcriptional GLUT4 activity at least in m. soleus and m. gastrocnemius. Protein analysis revealed that expression of GLUT4 protein content was in particular enhanced in membrane fractions suggesting an increased glucose transport capacity of muscles after strength training [6].

Finally, a mouse obesity model was used to evaluate the potentially beneficial effects of both training models on glucose homeostasis. Glucose intolerance due to insulin resistance is a common phenomenon after consumption of high fat diet in mice and rats [35] and an important step in the development of diabetes and cardiovascular disease [36], [37]. Thus, interventions which increase insulin sensitivity constitute important therapeutic strategies to improve metabolic dysfunction. Consumption of a high fat diet for two months induced significant increase of glucose intolerance as indicated by enhanced serum glucose levels after glucose loading. Fat consumption together with exercise training was followed by an improvement of glucose handling, which was similar for both exercise types. These results agree nicely with previous studies in patients with type II diabetes which could demonstrate regulating effects of both strength as well as endurance exercise on glucose metabolism [35], [36].Thereby, the long term effects of endurance training and strength training as indicated by HbA1c measurements were in the same range [36], [37].

Analysis of GLUT4 expression in muscles from high-fat-diet groups indicated again a preferential transcriptional regulation of GLUT4 mRNA after strength training. However, increased protein levels in both cytosolic as well as membrane fractions were found only by trend under the pathophysiological conditions. A reduction of mRNA expression after endurance training in hfET mice was conflictive to prior findings and our expectations [37], [38]. Nevertheless, the strength training model proves as a suitable approach to improve glucose handling which seems to be mediated by other signaling mechanisms than endurance training and should be elucidated in subsequent studies.

### Conclusions

Taken together, the current study evaluated a mouse model of isometric strength training which demonstrated specific adaptations to strength exercise with respect to performance parameters and muscular adaptations. Furthermore, this model could be distinguished from an endurance training model since the adaptations after both training regimes showed only a marginal overlapping. The isometric strength training model seems to be relevant also in a therapeutical context as it improves glucose handling in a pathophysiological diabetes model. Taken together, further studies using the strength training model seem to be useful in order to get more insights into the effects of strength training on a cellular and molecular level.
